# Simultaneously Quantifying Both Young’s Modulus and Specific Membrane Capacitance of Bladder Cancer Cells with Different Metastatic Potential

**DOI:** 10.3390/mi11030249

**Published:** 2020-02-27

**Authors:** Na Liu, Mengying Leng, Tao Yue, Liang Dong, Yuanyuan Liu, Yan Peng, Huayan Pu, Shaorong Xie, Jun Luo

**Affiliations:** 1 School of Mechatronics Engineering and Automation, Shanghai University, Shanghai 200444, China; liuna_sia@shu.edu.cn (N.L.); yl1566@shu.edu.cn (M.L.); yuanyuan_liu@shu.edu.cn (Y.L.); pengyan@shu.edu.cn (Y.P.); phygood_2001@shu.edu.cn (H.P.); srxie@shu.edu.cn (S.X.); luojun@shu.edu.cn (J.L.); 2 Shanghai Institute of Intelligent Science and Technology, Tongji University, Shanghai 200092, China; 3 School of Medicine, Shanghai Jiao Tong University, Shanghai 200240, China; drdongliang@126.com

**Keywords:** single cell analysis, bladder cancer, micropipette aspiration, Young’s modulus, specific membrane capacitance

## Abstract

Both Young’s modulus and specific membrane capacitance (SMC) are two important physical parameters for characterizing cell status. In this paper, we utilized a thin-neck-micropipette aspiration system to simultaneously quantify Young’s modulus and SMC value of six types of cell lines in different progression grades, which include four grades from the lowest metastatic potential G1 to the highest potential G4. We investigated how these two physical properties possess heterogeneities in bladder cancer cells with different grades and what roles they might play in grading bladder cancer. The characterization results of these cells of different cancer grades is linearly correlated with the cancer grades, showing that the Young’s modulus is negatively linearly correlated with bladder cancer grades, while SMC shows a positive linear correlation. Furthermore, the combination of these two physical properties on a scatter diagram clearly shows the cell groups with different cancer grades, which means that this combination could be a potential tumor grading marker to identify cancer cells with different metastatic potential.

## 1. Introduction

Bladder cancer is the fourth commonest cancer in men and the 11th commonest cancer in women [1,2]. In general, a high rate (70–80%) of bladder cancer is non-muscle-invasive bladder cancer (NMIBC) and a low rate (20–30%) is muscle-invasive bladder cancer (MIBC). Although patients with non-muscle-invasive bladder cancer (NMIBC) rarely die initially, there is a high recurrence rate of 50–70% and a high rate of 30% to progress into muscle-invasive bladder cancer (MIBC) [3,4,5]. For patients with NMIBC, the risk of disease recurrence (50–90%) after five years is directly proportional to the progression grades, and 10–30% will progress to invasive carcinoma within five years [3]. From the viewpoint of therapeutics and surveillance, it is significant to evaluate the progression grade in bladder cancer diagnoses.

In 1973, World Health Organization (WHO) adopted a numerical grading system to grade urothelial carcinoma from I to III according to epithelial abnormalities and cellular anaplasia [6]. Subsequently, the urothelial carcinoma grading system was continually modified. In 2004, WHO classified urothelial carcinoma as low or high grade based on architectural and cytological atypia [3,7,8]. However, these three grading systems are all based on morphological characteristics such as tissue structure (i.e., cell numbers, cell alignment, etc.) and cell morphology (i.e., cell size, nuclear size and shape, mitosis, and chromatin and nuclear fission, etc.). Grading bladder cancer based on these complicated morphological characteristics is often judged by subjective sense rather than quantitative analysis, so the grading results are highly decided by the level of pathologists [7]. An objective grading method based on quantitative characteristics is highly desirable for avoiding misdiagnosis caused by ambiguous subjectivity.

Cellular physical properties including Young’s modulus and specific membrane capacitance (SMC) are efficient biomarkers for charactering cell status [9,10,11,12,13,14,15,16]. During the process of healthy cells transforming into cancerous cells as well as the cancer cell progress, both the membrane and the cytoskeleton of cells undergo a series of changes [17,18,19,20]. These changes can lead the changes of cellular Young’s modulus and SMC value. A large number of reports have demonstrated that cancer cells possess lower Young’s modulus and larger SMC values than normal cells [21,22,23,24,25,26], which suggest that both Young’s modulus and SMC value are potential characteristics for grading bladder cancer. Several researchers have also tried to understand how physical property differences exist among the bladder cancer cells with different progression grades. For example, Coughlin et al. showed that bladder cancer cells with higher metastatic potential are softer than bladder cancer cells with lower metastatic potential by characterizing the deformation ability of 253J and 253JB5 cells using magnetic twisting cytometry [27]. Liu et al. reported that bladder cancer cell at higher grades possess larger SMC values and lower Young’s modulus than cancer cells at lower grades, by using two independent techniques of microfluidic and atomic force microscope (AFM) to measure RT4 and T24 cells [28]. Abidine et al. also observed that cellular elastic modulus is associated with cells’ invasion ability by measuring the elastic modulus of RT112, T24, and J82 cells using AFM [29]. However, these studies only measured either mechanical or electrical properties of cells due to the limits of the adopted tools. Additionally, only two or three types of bladder cancer cells were measured in these reports, which made it difficult to reach a consensus about how the electrical and mechanical properties of bladder cancer cells correlate with their progression grades [30]. In this paper, we applied a thin-neck-micropipette aspiration system that can simultaneously measure both SMC value and Young’s modulus of single cells to characterize six types of bladder cancer cells with four different grades. The results showed that there was a linear negative correlation between the Young’s modulus and the grade of bladder cancer (*R* = –0.9094), and a linear positive correlation between SMC and grade of bladder cancer (*R* = 0.8538), which demonstrated that quantifying cells’ physical properties is an alternative approach for grading bladder cancer.

## 2. Materials and Methods

### 2.1. An Aspiration System for Simultaneously Quantifying Cells’ Specific Membrane Capacitance (SMC) and Young’s Modulus

**Figure 1:**
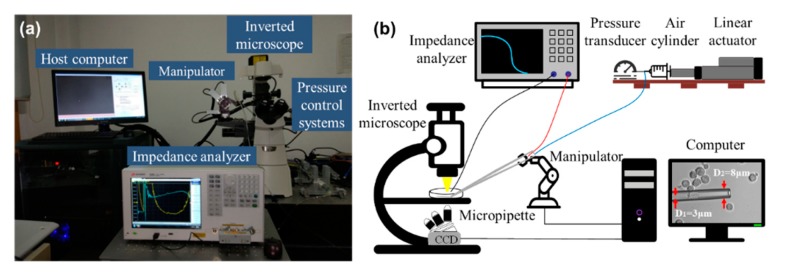
Setup of the whole system showing its key components including the micromanipulator, pressure controller, and the impedance analyzer. (**a**) A photo of the micropipette system; (**b**) schematic of the micropipette system.

We developed an aspiration system for simultaneously quantifying SMC and the viscoelasticity of single cells. As shown in Figure 1, it consists of an inverted microscope (Nikon ECLIPSE Ti-S, Nikon, Tokyo, Japan), a CCD camera (XC-555, Sony, Tokyo, Japan), an XY motorized stage (ProScan II, Prior Scientific Instruments Ltd., Cambridge, UK), a motorized micromanipulator (Mc1000e, Siskiyou, San Diego, CA, USA), a syringe-type pressure control system (customized by our lab), an impedance analyzer (KEYSIGHTE4990A, Keysight Technologies Inc., Santa Rosa, CA, USA), and a host computer (Lenovo Workstation, Beijing, China). Here, this system was improved to be capable of simultaneously measuring the SMC and Young’s modulus of a cell based on reprogramming the pressure control model.

A thin-neck-micropipette was used to aspirate the cells. The micropipette was made from a borosilicate glass tube with an inner diameter of 0.75 mm and an outer diameter of 1 mm. A thin neck was fabricated near the top, which could hold the cell and prevent it from becoming sucked too deeply into the micropipette. The feedback-based pressure control system could achieve a pressure range from –2500 to 2500 Pa. As shown in Figure 1b, the inner diameter of the thin neck structure (*D*_1_) was about 3 μm, and the inner diameter of the micropipette mouth (*D*_2_) was about 8 μm. 

### 2.2. The Aspiration Procedures for Quantifying Physical Properties of Cells

**Figure 2:**
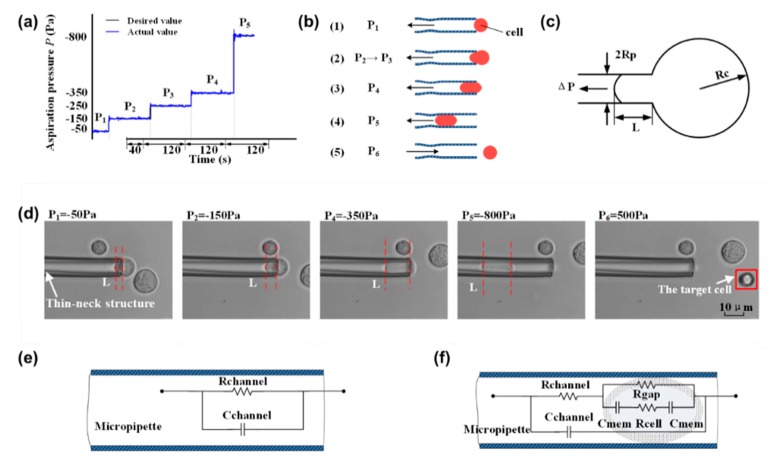
The aspiration procedures for quantifying physical properties of cells. (**a**) The distribution of negative pressure in the experiment for aspirating cells; (**b**) schematic diagram of the dynamic process of the micropipette attracting an individual cell; (**c**) homogenous half-space model schematic diagram; (**d**) a micrograph of a single cell entering the thin-neck-micropipette; (**e**) equivalent circuit model of the micropipette when no cells are aspirated; (**f**) equivalent circuit model of micropipette when there are aspirated cells in the micropipette.

To quantify the physical properties of cells, a cell was aspirated into a micropipette by controlling the pressure. As shown in Figure 2a,b, the process of cell aspiration was divided into four steps, including cell capture, Young’s modulus measurement, SMC value measurement, and cell release. For cell capture, a pressure of *P*_1_ (−50 Pa) was applied to hold the cell for 40 s. Then, the pressure was increased with a step of –100 Pa to *P*_2_ (−150 pa), and the cell was slowly squeezed into the micropipette. *P*_2_ was held for 120 s to ensure the cell stopped, and the aspirated length of the cell was recorded. The aspirated length was defined as the distance between the front end of the cell and the mouth of the constriction channel. The aspiration process was repeated by increasing *P*_2_ to *P*_3_ and *P*_3_ to *P*_4_. The Young’s modulus was calculated according to the recorded aspirated length and the corresponding pressure. For the SMC characterization, a large negative pressure *P*_5_ (−800 pa) was used to completely aspirate the cells into the pipette. Once the cell was trapped by the thin neck, the impedance spectrum was recorded in a frequency range of 102–106 Hz. Finally, a pressure of *P*_6_ (500 Pa) was applied to squeeze the cell out from the micropipette. Figure 2d shows the status of the cell under different aspirate pressures, where *L* represents the aspirated cell length.

### 2.3. Characterization of Single Cell Young’s Modulus

As shown in Figure 2c, when a sucking pressure Δ*P* was applied to aspirate a cell using a micropipette, a portion of the cell was aspirated into the micropipette. A homogenous half-space elastic model was applied for the characterization of Young’s modulus from the experimental data [31].

(1) $$\Delta P=\frac{2\pi }{3\phi }E\frac{L}{R_{P}}$$

where *L* refers to the aspiration length (the distance between the front end of the cell and the mouth of the constriction channel); Δ*P* is the aspiration pressure; *R_p_* is the inner diameter of the micropipette; *E* represents the Young’s modulus of the cell; and *ϕ* denotes the wall function, which is determined by the material and size of the microtubules; *ϕ* has a typical value of 2.1 [32].

### 2.4. Characterization of Single Cell SMC Value

A “single shell” model was employed to describe the electrical properties of the cell. Figure 2e shows the equivalent circuit model when there is no cell aspirated into the micropipette. The whole impedance *Z_e_* can be described as

(2) $$Z_{e}=\frac{1}{\frac{1}{R_{channel}}+j\omega C_{channel}}$$

where *R_channel_* and *C_channel_* represent the resistance and capacitance of the whole micropipette, respectively, and *ω* represents the angular frequency of the AC signal.

When the cell is squeezed into the micropipette, as shown in Figure 2f, the whole impedance *Z_p_* can be described as follows:

(3) $$Z_{p}=\frac{1}{\frac{1}{Z_{cp}+R_{channel}}+j\omega C_{channel}}$$

where *Z_cp_* can be described as

(4) $$Z_{cp}=\frac{\left(R_{c}+2\frac{1}{j\omega C_{mem}}\right)R_{gap}}{\left(R_{c}+2\frac{1}{j\omega C_{mem}}\right)+R_{gap}}$$

where *Z_cp_* is the equivalent circuit impedance of the whole cell; *R_c_* is the cell dielectric resistance; *R_gap_* is the leakage resistance; and *C_mem_* represents the membrane capacitance. The value can be determined by fitting the impedance spectrum using the above equations; the SMC value, defined as the capacitance per unit membrane area, can be calculated according to Equation (5).

(5) $$SMC=\frac{C_{mem}}{2\pi r_{c}^{2}}$$

### 2.5. Cell Preparation

Six types of bladder urothelial carcinoma (UC) cell lines were measured in this paper, including Grade 1 (G1) NMI transitional cell papilloma RT4 and SW780; G2 MI lines 5637; G3 MI lines T24 and J82; and G4 MI transitional cell carcinoma 253J. The cell lines were obtained from the Clinical Stem Cell Research Center of Shanghai Renji Hospital. The information of the former 5 cell lines was obtained from American Type Culture Collection (ATCC), and the 253J cell line was found in the KCLB (Korean Cell Line Bank). Regarding 253J, half of them came from female patients and the other half from male patients, which indicated that Young’s modulus value and SMC value are not affected by gender.

Before the experiments, cells were cultured in the incubator at a temperature of 37 °C, relative humidity of 100° and carbon dioxide concentration of 5%. Culture medium is Dulbecco’s modified Eagles medium (DMEM) supplemented with 1% penicillin and 10% fetal bovine serum (HyClone, GE Healthcare Bio-Sciences, Pittsburgh, PA, USA). For the physical properties quantification, all types of cells were digested from the 25-cm^2^ culture flasks and resuspended into 2-mL PBS solutions, respectively. Fifteen cells from each type of cell were measured. In experiments, cells were completely exposed to the atmosphere with room temperature 25 °C. As cells are highly susceptible to pollution leading to denaturation and inactivation, we ensured the disinfection environment in the laboratory and controlled the experiment operation time of a single group of cells to 8 min to minimize the impact of environmental changes.

### 2.6. Data Acquisition and Analysis

Micrograph images were taken using an inverted microscope (Nikon Ti-E, Japan). Multiple images of each cell type were obtained at different times, and the images were processed, respectively, by ImageJ (version 1.47, NIH, Bethesda, MD, USA) to calculate the cell suction length and the inner diameter of the micropipette, and make a typical comparison of different types of cells. Both Young’s modulus and SMC values were presented as the mean ± standard error of the mean unless stated and were plotted as boxplots. Means and standard error of the mean were calculated using Origin Pro (OriginLab Corporation, Northampton, MA, USA). Statistical analysis was applied for determining the statistical significance (P) between samples from different experimental groups of equal variance. Statistics were performed using the unpaired t-test (NS, not significant; * *p* < 0.05; ** *p* < 0.01; *** *p* < 0.001) by using GraphPad Software (GraphPad Prism 8.0.2, San Diego, CA, USA), as well as the linear regression.

## 3. Results and Discussion

### 3.1. Young’s Modulus and SMC Value of Single Bladder Cancer Cells are Linearly Correlated with the Cancer Grades

**Figure 3:**
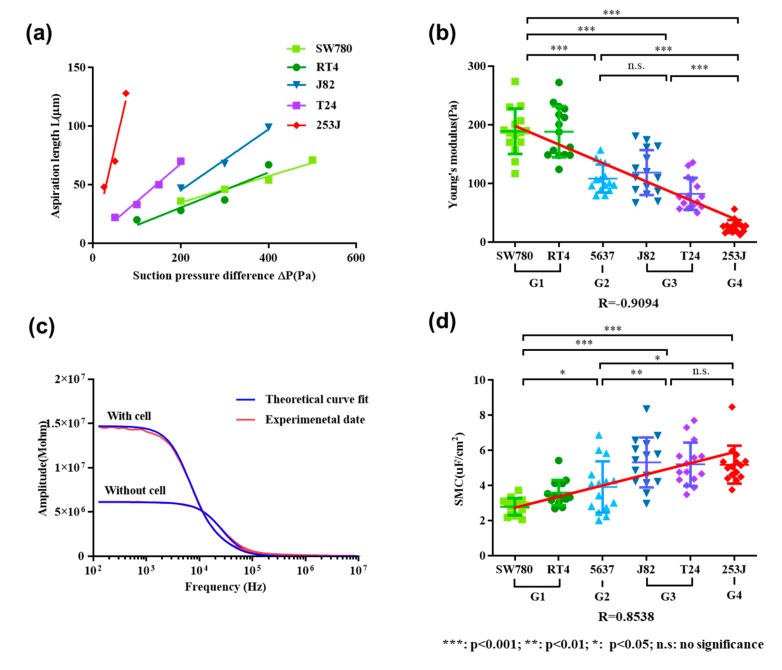
The Young’s modulus is negatively linearly correlated with bladder cancer grades, while specific membrane capacitance (SMC) showed a positive linear correlation. (**a**) Diagram of adsorption pressure relative to cell length; (**b**) Young’s modulus of six types of cell lines; (**c**) impedance spectra in no-load and cellular states of T24; (**d**) SMC value of six types of cell lines.

Young’s modulus of cells was calculated according to the relationship between sucking pressure and the aspiration length. According to Equation (1), the relationship between the aspiration length and the adsorption pressure can be described as Equation (6). Figure 3a shows the functional relationship represented by Equation (6), which indicates the relationship between the adsorption pressure and the aspiration length of some cells.

(6) $$L=\frac{3\phi R_{P}\Delta P}{2\pi }\frac{1}{E}$$

The Young’s modulus of cells can be calculated by the cell adsorption length *L* of the micropipette, adsorption pressure Δ*P*, and the inner diameter *R_p_* obtained from image processing. The higher the slope of the line, the smaller the Young’s modulus of the cell.

The average and deviation of Young’s modulus is illustrated in Figure 3b. The Young’s modulus was quantified to be 188.92 ± 38.63 Pa for SW780 cells, 188.36 ± 43.62 Pa for RT4 cells, 108.48 ± 23.97 Pa for 5637 cells, 118.57 ± 38.25 Pa for J82 cells, 82.28 ± 27.35 Pa for T24 cells, and 26.65 ± 10.94 Pa for 253J cells. It shows that the Young’s modulus of SW780 (G1) and RT4 (G1) were apparently higher than the other four kinds of cell lines, while the Young’s modulus of 253J (G4) was the lowest. According to the t-test, there was no significant difference in Young’s modulus between the two types of cells at G2 (5637 cells) and G3 (J82 cells and T24 cells), respectively, while there was a significant difference between the two types of cells at other grades. Using linear correlation analysis, a correlation coefficient of –0.9094 was obtained, which means there was a negative linear correlation between Young’s modulus and the grade of bladder cancer cells.

Figure 3c shows two typical impedance spectrums when there is a cell or no cell inside the micropipette, which were used to extract the SMC value of the aspirated cell. The extracted SMC values of six types of cells are shown in Figure 3d, which are 2.79 ± 0.48 μF/cm^2^ for SW780 cells, 3.60 ± 0.71 μF/cm^2^ for RT4 cells, 3.92 ± 1.46 μF/cm^2^ for 5637 cells, 5.32 ± 1.42 μF/cm^2^ for J82 cells, 5.21 ± 1.24 μF/cm^2^ for T24 cells, and 5.19 ± 1.08 μF/cm^2^ for 253J cells. It shows that the SMC value of SW780 (G1) and RT4 (G1) was significantly lower than the other four types of cell lines. With the increase of grade, SMC value had a tendency to increase gradually. On the basis of the t-test, except that there was no significant difference in SMC values between G3 (J82 cells and T24 cells) and G4 (253J cells), there were significant differences between other grades. A linear correlation coefficient R of 0.8538 was obtained, demonstrating that the SMC values were positively correlated with the grade of bladder cancer cells.

### 3.2. Combination of Young’s Modulus and SMC Value Could Be A Potential Tumor Grading Marker to Identify Cancer Cells With Different Metastatic Potential

**Figure 4:**
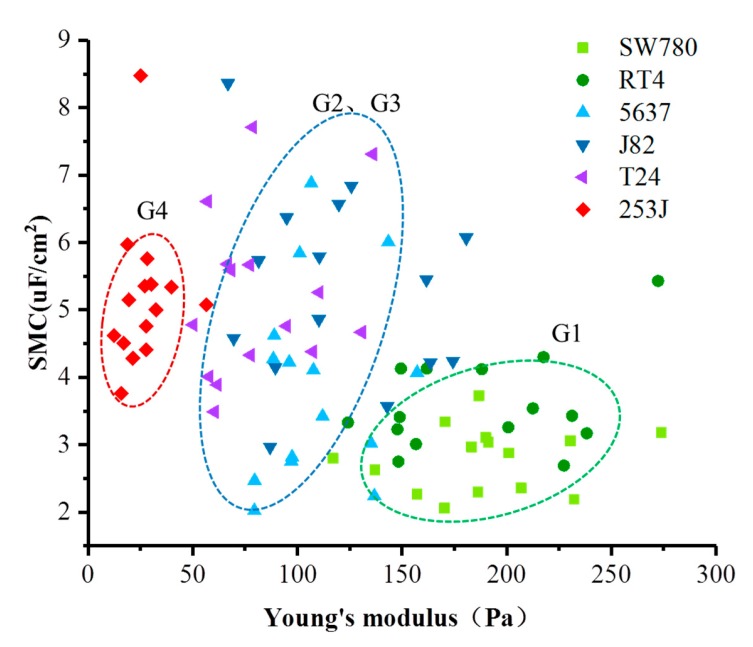
The scatter diagram of all data points. Points marked by different colors and shapes come from different cell lines. Almost all of the data points are divided into three groups: the blue oval area, the red oval area, and the green oval area.

In order to extend the one-dimensional correlation of Young’s modulus and SMC with cancer grades into a two-dimensional fashion, a scatter diagram of all data points was drawn, as shown in Figure 4, which provided clearer group information of these cancer cells according to their progression stages and metastatic potential. The data points of different cell lines were marked by different colors and shapes. Based on the combination of two physical properties, the data points fell into three categories: the points of 253J cell lines were mainly contained in the green oval area, which is G4; the points of T24, J82, and 5637 cell lines were mainly contained in the blue oval area, which are G2 and G3; the points of SW780 and RT4 cell lines were mainly contained in the red oval area, which is G1. These results showed that there were significant differences in physical characteristics between different grades of bladder cancer, and the integration of different physical properties could be a more solid marker to identify these cells with different cancer grades.

## 4. Conclusions

Cancer progression is associated with abnormal biophysical properties of cells. The measurement of these biophysical properties provides a new perspective for studying cancer. In this paper, a micropipette aspiration system, which is able to simultaneously quantify both the Young’s modulus and SMC value of a single cell, was utilized to study how the physical properties correlated with the bladder cancer grades. Six types of bladder cancer cell lines (SW780 (G1), RT4 (G1), 5637(G2), J82 (G3), T24 (G3), and 253J (G4)) corresponding to four different metastatic potentials were measured. The results showed that there was a linear negative correlation between Young’s modulus and grade of bladder cancer (*R* = –0.9094), and a linear positive correlation between SMC and grade of bladder cancer (*R* = 0.8538). Based on the scatter diagram, we demonstrated that cells with different metastatic potential are distinguishable based on their SMC values and Young’s modulus. Such simultaneous measurements of Young’s modulus and SMC value could provide an unbiased marker of cancer grades and metastatic potential that may help us understand cancer progression and establish a faster and easier bladder cancer diagnosis approach.
